# Combining high dose therapy, bilateral motor priming, and vagus nerve stimulation to treat the hemiparetic upper limb in chronic stroke survivors: a perspective on enhancing recovery

**DOI:** 10.3389/fneur.2023.1182561

**Published:** 2023-06-28

**Authors:** Erin C. King, Elizabeth Pedi, Mary Ellen Stoykov, Daniel M. Corcos, Sebastian Urday

**Affiliations:** ^1^Northwestern University, Evanston, IL, United States; ^2^Physical Therapy & Human Movement Sciences, Feinberg School of Medicine, Northwestern University, Chicago, IL, United States; ^3^Shirley Ryan AbilityLab, Chicago, IL, United States; ^4^Department of Physical Medicine and Rehabilitation, Feinberg School of Medicine, Northwestern University, Chicago, IL, United States; ^5^The Ken & Ruth Davee Department of Neurology, Feinberg School of Medicine, Northwestern University, Chicago, IL, United States

**Keywords:** stroke, rehabilitation, priming, task specific training, vagus nerve stimulation

## Abstract

Stroke is a leading cause of disability worldwide and upper limb hemiparesis is the most common post-stroke disability. Recent studies suggest that clinically significant motor recovery is possible in chronic stroke survivors with severe impairment of the upper limb. Three promising strategies that have been investigated are (1) high dose rehabilitation therapy (2) bilateral motor priming and (3) vagus nerve stimulation. We propose that the future of effective and efficient upper limb rehabilitation will likely require a combination of these approaches.

## Introduction

Today, there are over 7 million stroke survivors in the United States alone (1). It is estimated that about 50% remain chronically disabled by residual post-stroke impairments (1). In contrast to the advances made in the treatment of acute stroke over the past decade, few breakthroughs have emerged to enhance neurological recovery in the chronic stage (>6 months) until very recently. As the number of stroke survivors continues to increase due to population growth and rising life expectancies, new strategies are needed to improve the quality and delivery of neurorehabilitation to people in the chronic phase of recovery.

Upper limb (UL) hemiparesis is the most common post-stroke impairment and is significantly associated with a loss of independence in activities of daily living, decreased quality of life, and increased risk of institutionalization (2, 3). As such, recovery of UL function has been a top priority target for stroke rehabilitation researchers, stroke survivors and healthcare professionals alike (4). A recent systematic review of rehabilitation interventions for the hemiplegic UL at acute, sub-acute and chronic stages reports an evidence base of 1,307 randomized controlled trials as of April 2021, with an average 300 RCTs added annually (5). Despite this large database, most large-scale chronic stroke trials targeting recovery of the UL have failed to find a difference between the experimental intervention and usual care (6–8). While there has been some debate concerning whether individuals with severe UL impairment can benefit from rehabilitation intervention (9), recent work has demonstrated that recovery for people with moderate–severe hemiparesis in the chronic phase is both possible and clinically significant (10–13).

The purpose of this perspective is to explore 3 treatment approaches that have shown promise to induce meaningful recovery in chronic stroke survivors with moderate-to-severe UL hemiparesis including (1) high doses of therapy (2) bilateral motor priming (BMP), and (3) vagus nerve stimulation (VNS). We aim to highlight the potential of BMP to enhance the effects of high dose UL therapy and suggest that, in combination with VNS, this approach may lead to improved functional outcomes for people with chronic UL impairments.

## Importance of dosing

### High dose

The dose of therapy can refer to the amount of time scheduled for therapy, the time spent in active practice, or the number of repetitions achieved within a session (14). Most often the dose is reported as the amount of time scheduled in therapy, but this frequently fails to accurately reflect the time spent in active practice and the quality of the active practice within a treatment session. By any definition, the optimal dose of therapy to bring about maximal recovery in the UL is yet to be determined. The consensus in human trials seems to be ‘more is better’ (15), however there have been mixed results regarding the efficacy of high dose (18–36 h) in several larger randomized controlled trials (6, 16–18). Recently, two studies found that 90 (10) and 300 h (11) of UL training in chronic stroke survivors led to significant improvement in motor impairments, suggesting that prior dose studies underestimated the amount of therapy needed to effect change.

These studies, led by Ward and McCabe respectively, found that large doses of therapy (90–300 h) were well-tolerated and led to significant improvement of motor deficits (10, 11). They enrolled patients with moderate-to-severe UL hemiparesis. Their findings support the view that increased hours of practice can lead to the re-learning of motor skills lost as a result of a stroke (19). Ward and colleagues (10) enrolled 224 chronic stroke survivors that received approximately 30 h of therapy each week over 3 weeks totaling 90 h. The intervention consisted of 2 daily sessions of physical and occupational therapy (4 h total) and another 2 h of any combination of the following: practice with a rehabilitation aid, robotic device, electric stimulation, and/or groupwork. This prospective observational study did not have a control group. The Fugl-Meyer Assessment of upper extremity motor recovery (FMUE) increased by a median change score of 9 points from pre- to follow-up, 6 months after treatment ended.

McCabe and colleagues reported similarly impressive results (11). Thirty-six participants with comparable baseline characteristics to Ward received 300 h of intervention: 5 h per day for 5 days per week, for 12 weeks. Participants were randomized to one of 3 treatment groups where 30% of participants’ treatment time was allocated to the application of (1) distal, (2) proximal, or (3) combined distal and proximal technologies (i.e., robotics training, functional electrical stimulation). The remaining 70% of treatment time was spent performing functional task practice guided by motor learning principles (11). The groups improved equally from pre- to post-intervention. Data were subsequently combined in further analyses, to determine if a mid-treatment plateau occurred at 150 h, or if participants benefited from continued therapy independent of group assignment. The analysis indicated that significant recovery occurred during the second half of the treatment timeline (20). The mean pre to post improvement on the FMUE was 9.8 points (20).

The Ward and McCabe studies demonstrate improvements approximately double in magnitude compared to prior studies and substantially exceeds the established clinically important difference (CID) for the FMUE defined as a range of 4.25–7.25 points in chronic stroke (21). Together, they highlight the potential of well-implemented, high dose neuro-rehabilitation protocols to produce meaningful improvements in patients with significant arm weakness in the chronic phase post-stroke.

### Dose delivery

In the context of the findings of Ward and McCabe, the amount of scheduled therapy in the United States (US) is far below what is needed to optimize recovery. A recent multi-site study (28 acute care hospitals) in the US tracked amount of therapy that patients received over 1 year post-stroke (22). The mean combined number of occupational (OT) and physical therapy (PT) sessions attended within a 12 month period post-stroke was just 31.7 (22). “Session” was defined as any active participation with a therapist lasting at least 1 min, and average duration was not reported. Using Medicare data, another study reported the combined total time spent in OT and PT in the first-year post-stroke as only 25 h (23). In the US, amount of therapy is driven by payer rather than patient needs. Rehabilitation services are the leading post-stroke care expense, with insurance caps often limiting therapy coverage and patient progress (24). Scheduling 90 or 300 h of rehabilitation like those seen in the Ward and McCabe studies is, thus, impossible in the current US healthcare system.

In addition to limited therapy hours, the dose of therapy when defined by the number of UL repetitions delivered during a single session, is also low. One observational study of post stroke standard of care rehabilitation services found that individuals complete just 32 functional movement repetitions of the UL per therapy visit (25). Minimal repetitions within a session fails to maximize the already limited number of hours a patient receives therapy. An investigation into the effects of delivering higher repetitions of a task or movement found that scaling from 100 to 300+ repetitions of the UL per hour for 32 h over 8 weeks had minimal, nonsignificant effects on outcomes (17). The authors speculate that they may have failed to see improvement in motor function as a product of increasing repetitions because the number of repetitions performed was still too low when compared to the number of movements required for recovery in the rodent model. They further argue that factors related to participant tolerance and abilities could limit the delivery of additional repetitions beyond 300. This study demonstrates that a therapeutic protocol with a focus on high repetitions alone is unlikely to induce significant change.

To achieve their respective results, Ward and McCabe provided interventions that included self-directed and therapist-led UL activities with a general goal of high repetitions of functional tasks (the exact number of repetitions was not reported). One way to interpret the data from these collective studies is to conclude that one needs at least of 90 h of therapy to see therapeutic benefit and improvement on the FMUE of 9–10 points. Another takeaway is that the therapy provided should aim to include a combination of customized, highly intensive, and progressive activities that challenge motor skill.

## Therapy adjuvants

### Bilateral motor priming

Promisingly, a recent smaller-scale study demonstrated that significant recovery for chronic UL hemiparesis *is possible* using fewer therapy hours if a comprehensive UL training protocol is preceded by motor priming (12). Several studies suggest that priming can be used as an adjuvant to rehabilitation therapy in chronic stroke survivors with moderate-to-severe UE hemiparesis (12, 26, 27). Priming is a neuromodulatory technique delivered prior to therapy and is designed to ready, or “prime” the brain to enhance motor recovery (28). Priming techniques include stimulation-based methods (e.g., transcranial direct current stimulation, repetitive transcranial magnetic stimulation), pharmacology-based priming, sensory based priming, and movement-based priming such as bilateral motor priming (28).

BMP uses the “Rocker” (Exsurgo Bilateral Priming Device, Auckland, NZ) to induce priming. Both hands are strapped into vertically oriented plates attached via a mechanical link. Prior to therapy, participants move both wrists in rhythmic, symmetrical wrist flexion and extension for 15 min at a frequency of 1 Hz with a goal of 900 repetitions. Participants do not need to have active flexion and extension of the affected hand, as the less affected arm drives the weaker one in in-phase bilateral symmetrical movement. Thus, precluding wrist contractures and severe spasticity, individuals at all levels of post-stroke motor recovery can use this priming method.

A study by Stoykov and colleagues showed that 7.5 h of BMP and 22.5 h of task-specific training (TST) over an intended duration of 5 weeks led to similar improvement as the previously discussed large-dose therapy trials (12). In each session, participants performed 15 min of BMP followed by 45 min of task-specific training under the direction of an occupational therapist. Following a 30–60 min break, this schedule was repeated. Median FMUE change scores for the BMP group were comparable to Ward, with a median increase of 11 points from pre- follow-up, 6 weeks after therapy ended (12). Although both the primed and unprimed (control) groups improved at post-treatment, both clinically and statistically significant between-group differences were greatest at follow up. Notably, the protocol was delivered in a third of the time (30 h) of the program by Ward and colleagues (90 h).

The causal mechanisms of BMP remain under investigation. When combined with different kinds of UL therapy, BMP appears to induce specific neurophysiological changes, namely, increased corticomotor excitability in the lesioned hemisphere and increased transcallosal inhibition from the lesioned hemisphere to the non-lesioned hemisphere (12, 26). This is significant because improvement in motor function after a stroke is linked to the reinstatement of balanced corticomotor excitability and transcallosal inhibition (29, 30).

BMP appears to amplify the positive effect of rehabilitation and is non-invasive, safe, and inexpensive to implement. In addition, when the dose (defined in hours) of rehabilitation that can be provided is limited, BMP may be particularly attractive as an adjunct. BMP has the potential for broad clinical application.

### Vagus nerve stimulation

Vagus nerve stimulation combined with upper limb training has demonstrated efficacy for treatment of the hemiparetic UL and holds great clinical potential (13, 31). In a large, multi-center randomized control trial, researchers paired VNS with an UL training protocol which focused on “active movements, task specificity, high number of repetitions, variable practice and active participant engagement” and compared outcomes to a group receiving sham stimulation and the same UL training protocol (13, 32). A trained therapist administered VNS pulses during each repetition of active movement. It was estimated that participants performed at least 300 movement repetitions during each session. Participants attended 90–120 min sessions 3 times a week for 6 weeks (18 sessions). Additionally, participants were instructed to perform a self-directed home exercise program (HEP) after first setting VNS to ON by swiping a magnet over the stimulator. The self-initiated VNS paired with the HEP continued after in-clinic therapy had ended, for up to 1 year.

Participants in the VNS group saw a significantly higher change in the FMUE from baseline to 1 day post treatment than the control group who received sham stimulation and the same UL treatment (mean change of 5.0 compared to 2.8) (13). Additionally, a clinically important difference, defined in this study as an increase of 6 or more points on the FMUE, occurred in more participants in the VNS group compared to the sham stimulation group 90 days after in-clinic treatment ended (47% versus 24%) (13).

A subsequent review and meta-analysis of the effect of VNS on UL recovery post-stroke further confirms that VNS paired with UL therapy produces significant, immediate, and long-term positive changes in stroke patients (31). These positive results led to recent approval of this rehabilitative modality by the U.S. Food and Drug Administration. As healthcare systems prepare to adopt VNS as a strategy to improve outcomes for stroke patients, it is important to consider the therapy which is delivered alongside stimulation.

Although the mechanisms underlying its efficacy are not yet fully known, it is thought that VNS may facilitate neuroplastic changes in the brain by increasing the release of neurotransmitters such as acetylcholine and norepinephrine in the cortex (33). When stimulation is paired with UL training, the enhanced neuroplasticity may support the rewiring of neural pathways associated with motor function and improve recovery post-stroke (33).

If VNS is paired with usual care therapy as it is currently delivered, the dose is arguably too low to achieve the gains demonstrated in the 2021 trial. To see benefit, patients will need to receive a minimum of 27–36 treatment hours over 6 weeks, post VNS implantation. As previously stated, the mean combined number of PT/OT sessions attended *in the entire year* following stroke is 31.7 in the U.S. VNS will fail to achieve the gains demonstrated in the 2021 trial if combined with this insufficient number of therapy sessions. There is therefore an urgent need to optimize dose delivery in terms of both hours scheduled and the rigor of UL training in the clinical setting to take advantage of the exciting study results above.

## Discussion

**Figure 1:**
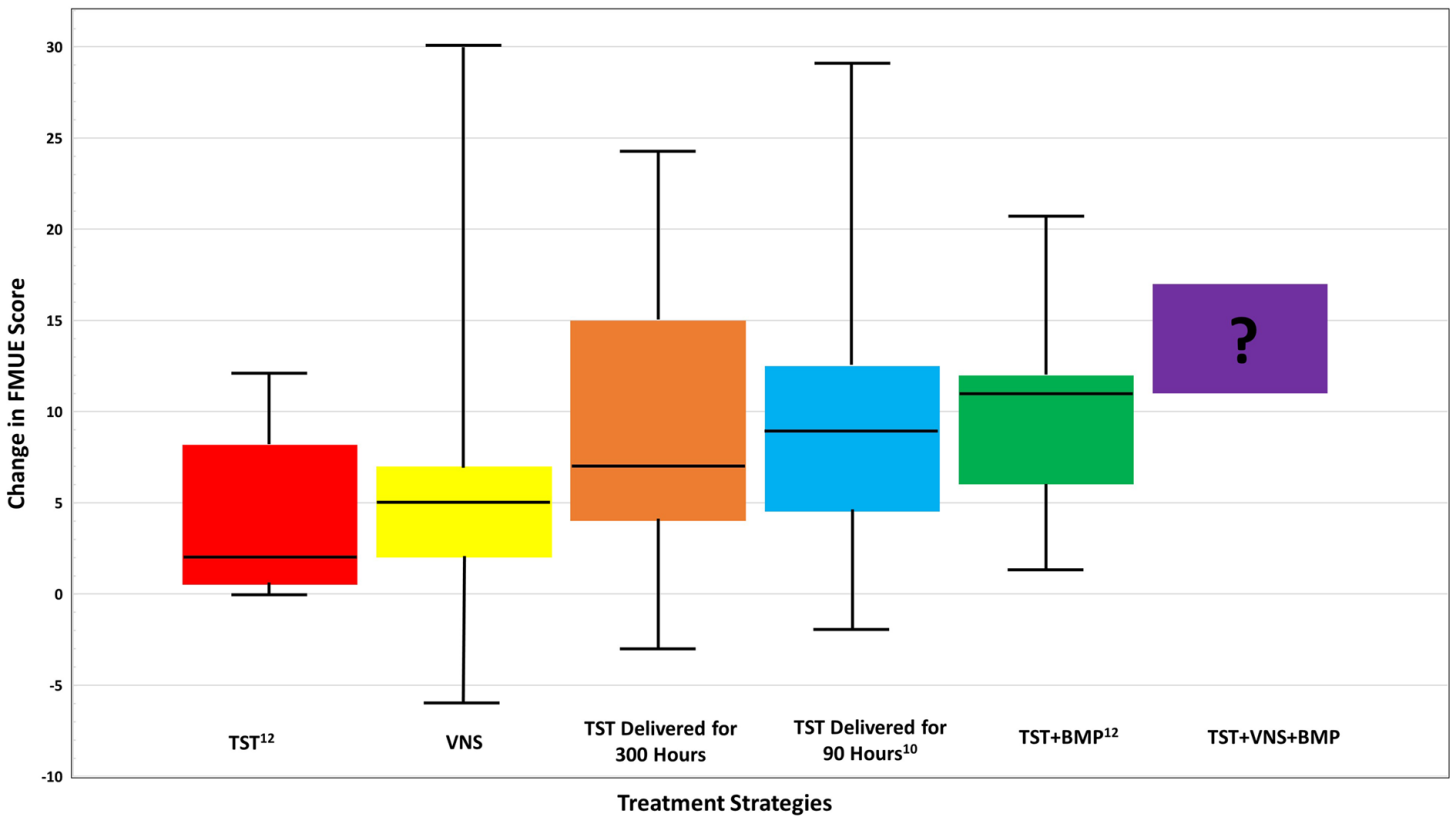
Comparison of median change in FMUE score across treatment strategies for chronic stroke, from baseline to follow up. Prospective functional improvement of combined TST + BMP + VNS is also shown. Boxplot whiskers denote maximum and minimum data points. TST, task specific training; BMP, bilateral motor priming; VNS, vagus nerve stimulation. Data received via email communications with J. Dawson, MD, and J. Daly, PhD in May 2023.

The above cited studies advance our understanding of key ingredients necessary to improve UL function in chronic stroke survivors including (1) increased hours of scheduled UL therapy (2) increased emphasis on skilled, progressive movement within the scheduled time (3) BMP and VNS to maximize the effects of an intensive UL therapy protocol. The data presented in Figure 1 outlines the median change in FMUE using the various strategies discussed above and the potential improvement if the strategies were combined. The data reported as median values was obtained through personal communication with the authors to enable an accurate comparison of results. The figure highlights possible improvement when TST is delivered alone, as shown in the Stoykov study published in 2020 (red box), in which the control group received only TST and still saw benefit (12). Promisingly, in multiple recent studies, variations of the TST protocol consistently demonstrated efficacy in improving UL motor function for all groups probably explaining why experimental interventions failed to produce between-group differences (7, 34, 35).

Further benefit is possible when a robust UL training protocol is paired with VNS (yellow box) with 27–36 h of an individualized, progressive UL protocol that induced a median change score of 5.0. Improved results occurred when 22.5 h of a TST UL protocol was paired with 7.5 h of BMP producing a median FMUE change score of 11 points pre- follow-up (green box) compared to 2.2 points in the control group (red box) (12). Both the McCabe and Ward studies included TST components and demonstrated a median change from pre to follow up of 7 (orange box) and 9 (blue box), respectively.

Given that both methods have proven safe and effective for people with chronic, severe UL impairment, it is worth considering whether BMP when paired with VNS and a robust UL protocol can produce additive effects for maximal functional improvement. The exact neural mechanisms of the effects of either BMP or VNS on post-stroke UL hemiparesis are not known. However, after 20 min of the mirror symmetric wrist movements (BMP), healthy participants demonstrated an increase in cortical excitability of the resting passive hemisphere up to 20 min post movement (36). This increase in excitability did not interfere with selective muscle activity needed for controlled movement. Thus, BMP (if provided immediately before VNS plus task specific training) may add to the subsequently timed neuromodulation plus task specific training to achieve an optimized outcome.

Based on principles of metaplasticity, the effects of one technique (BMP or VNS) could reduce the plasticity-inducing effects of the other (37). Although unlikely, the possibility cannot be ruled out at this time. Another important question is how priming mechanisms may change over time after repeated sessions. These and other questions require well-designed neurophysiologic studies to move the field forward.

The potential improvement possible when the three strategies are combined is shown in Figure 1 as the purple box denoted with a question mark. Collectively, these studies clearly suggest that future research should explore how to combine TST with BMP and VNS to produce meaningful improvements that will give hope to patients with chronic hemiparetic stroke.

While the results of these studies are promising, realistic implementation into U.S. clinical practice will require thoughtful consideration. It is necessary to combine and deliver interventions with proven efficacy that are also feasible within the time constraints of the U.S. healthcare system which is unlikely to change drastically in the coming years. One strategy to deliver high dose, rigorous UL therapy is to combine face-to-face therapy sessions with supplemental exercise and education using smartphone apps, which has been effective when used in other diagnoses (38, 39). The VNS protocol took advantage of a targeted home exercise program. This would potentially reduce the number of in-person therapy sessions required, while allowing the patient to complete more skilled UL repetitions. In summary, the rehabilitation potential of individuals with chronic, moderate–severe UL hemiparesis is noticeably brighter than it was 6 years ago. We look forward to future advances in the delivery of TST combined with BMP and VNS or both.

## Data availability statement

The original contributions presented in the study are included in the article/supplementary material, further inquiries can be directed to the corresponding author.

## Author contributions

EK, EP, and SU drafted the manuscript and figures. All authors contributed to the article and approved the submitted version.

## Funding

This research is supported by National Institutes of Health (1RO1HD091492). EK is supported by 1F31HD111318-01. EP is supported by T32HD101395.

## Conflict of interest

The authors declare that the research was conducted in the absence of any commercial or financial relationships that could be construed as a potential conflict of interest.

## Publisher’s note

All claims expressed in this article are solely those of the authors and do not necessarily represent those of their affiliated organizations, or those of the publisher, the editors and the reviewers. Any product that may be evaluated in this article, or claim that may be made by its manufacturer, is not guaranteed or endorsed by the publisher.

## References

[1] Tsao CW Aday AW Almarzooq ZI Alonso A Beaton AZ Bittencourt MS. Heart disease and stroke statistics-2022 update: a report from the American Heart Association. Circulation. (2022) 145:e153–639. doi: 10.1161/CIR.0000000000001052, PMID: 35078371

[2] Winstein CJ Stein J Arena R Bates B Cherney LR Cramer SC. Guidelines for adult stroke rehabilitation and recovery: a guideline for healthcare professionals from the American Heart Association/American Stroke Association. Stroke. (2016) 47:e98–e169. doi: 10.1161/STR.0000000000000098, PMID: 27145936

[3] Sathian K Buxbaum LJ Cohen LG Krakauer JW Lang CE Corbetta M. Neurological principles and rehabilitation of action disorders: common clinical deficits. Neurorehabil Neural Repair. (2011) 25:21S–32S. doi: 10.1177/1545968311410941, PMID: 21613535 PMC4139495

[4] Pollock A St George B Fenton M Firkins L. Top 10 research priorities relating to life after stroke – consensus from stroke survivors, caregivers, and health professionals. Int J Stroke. (2014) 9:313–20. doi: 10.1111/j.1747-4949.2012.00942.x, PMID: 23227818

[5] Saikaley M McIntyre A Pauli G Teasell R. A systematic review of randomized controlled trial characteristics for interventions to improve upper extremity motor recovery post stroke. Top Stroke Rehabil. (2022) 30:323–32. doi: 10.1080/10749357.2022.2035578 35156561

[6] Winstein CJ Wolf SL Dromerick AW Lane CJ Nelsen MA Lewthwaite R. Effect of a task-oriented rehabilitation program on upper extremity recovery following motor stroke: the ICARE randomized clinical trial. JAMA. (2016) 315:571–81. doi: 10.1001/jama.2016.0276, PMID: 26864411 PMC4795962

[7] Levy RM Harvey RL Kissela BM Winstein CJ Lutsep HL Parrish TB. Epidural electrical stimulation for stroke rehabilitation: results of the prospective, multicenter, randomized. Single-Blinded Everest Trial Neurorehabil Neural Repair. (2016) 30:107–19. doi: 10.1177/1545968315575613, PMID: 25748452

[8] Winstein C Varghese R. Been there, done that, so what’s next for arm and hand rehabilitation in stroke? NeuroRehabilitation. (2018) 43:3–18., PMID: 29991146 10.3233/NRE-172412

[9] Pereira S Graham JR Shahabaz A. Rehabilitation of individuals with severe stroke: synthesis of best evidence and challenges in implementation. Top Stroke Rehabil. (2012) 19:122–31. doi: 10.1310/tsr1902-122, PMID: 22436360

[10] Ward NS Brander F Kelly K. Intensive upper limb neurorehabilitation in chronic stroke: outcomes from the queen square programme. J Neurol Neurosurg Psychiatry. (2019) 90:498–506. doi: 10.1136/jnnp-2018-319954, PMID: 30770457

[11] McCabe J Monkiewicz M Holcomb J Pundik S Daly JJ. Comparison of robotics, functional electrical stimulation, and motor learning methods for treatment of persistent upper extremity dysfunction after stroke: a randomized controlled trial. Arch Phys Med Rehabil. (2015) 96:981–90. doi: 10.1016/j.apmr.2014.10.022, PMID: 25461822

[12] Stoykov ME King E David FJ Vatinno A Fogg L Corcos DM. Bilateral motor priming for post stroke upper extremity hemiparesis: a randomized pilot study. Restor Neurol Neurosci. (2020) 38:11–22. doi: 10.3233/RNN-190943, PMID: 31609714 PMC7205167

[13] Dawson J Liu CY Francisco GE Cramer SC Wolf SL Dixit A. Vagus nerve stimulation paired with rehabilitation for upper limb motor function after ischaemic stroke (VNS-REHAB): a randomised, blinded, pivotal, device trial. Lancet. (2021) 397:1545–53. doi: 10.1016/S0140-6736(21)00475-X, PMID: 33894832 PMC8862193

[14] Lohse KR Lang CE Boyd LA. Is more better? Using metadata to explore dose-response relationships in stroke rehabilitation. Stroke. (2014) 45:2053–8. doi: 10.1161/STROKEAHA.114.004695, PMID: 24867924 PMC4071164

[15] Schneider EJ Lannin NA Ada L Schmidt J. Increasing the amount of usual rehabilitation improves activity after stroke: a systematic review. J Physiother. (2016) 62:182–7. doi: 10.1016/j.jphys.2016.08.006, PMID: 27637769

[16] Lo AC Guarino PD Richards LG Haselkorn JK Wittenberg GF Federman DG. Robot-assisted therapy for long-term upper-limb impairment after stroke. N Engl J Med. (2010) 362:1772–83. doi: 10.1056/NEJMoa0911341, PMID: 20400552 PMC5592692

[17] Lang CE Strube MJ Bland MD Waddell KJ Cherry-Allen KM Nudo RJ. Dose response of task-specific upper limb training in people at least 6 months poststroke: a phase II, single-blind, randomized, controlled trial. Ann Neurol. (2016) 80:342–54. doi: 10.1002/ana.24734, PMID: 27447365 PMC5016233

[18] Klamroth-Marganska V Blanco J Campen K Curt A Dietz V Ettlin T. Three-dimensional, task-specific robot therapy of the arm after stroke: a multicentre, parallel-group randomised trial. Lancet Neurol. (2014) 13:159–66. doi: 10.1016/S1474-4422(13)70305-3, PMID: 24382580

[19] Krakauer JW. Motor learning: its relevance to stroke recovery and neurorehabilitation. Curr Opin Neurol. (2006) 19:84–90. doi: 10.1097/01.wco.0000200544.29915.cc 16415682

[20] Daly JJ McCabe JP Holcomb J Monkiewicz M Gansen J Pundik S. Long-dose intensive therapy is necessary for strong, clinically significant, upper limb functional gains and retained gains in severe/moderate chronic stroke. Neurorehabil Neural Repair. (2019) 33:523–37. doi: 10.1177/1545968319846120, PMID: 31131743 PMC6625035

[21] Page SJ Fulk GD Boyne P. Clinically important differences for the upper-extremity Fugl–Meyer scale in people with minimal to moderate impairment due to chronic stroke. Phys Ther. (2012) 92:791–8. doi: 10.2522/ptj.20110009, PMID: 22282773

[22] Young BM Holman EA Cramer SC Investigators SS. Rehabilitation therapy doses are low after stroke and predicted by clinical factors. Stroke. (2023) 54:831–9. doi: 10.1161/STROKEAHA.122.041098 36734234 PMC9992003

[23] Skolarus LE Feng C Burke JF. No racial difference in rehabilitation therapy across all post-acute care settings in the year following a stroke. Stroke. (2017) 48:3329–35. doi: 10.1161/STROKEAHA.117.017290 29089456 PMC5705290

[24] Godwin KM Wasserman J Ostwald SK. Cost associated with stroke: outpatient rehabilitative services and medication. Top Stroke Rehabil. (2011) 18:676–84. doi: 10.1310/tsr18s01-676, PMID: 22120036

[25] Lang CE Macdonald JR Reisman DS. Observation of amounts of movement practice provided during stroke rehabilitation. Arch Phys Med Rehabil. (2009) 90:1692–8. doi: 10.1016/j.apmr.2009.04.005, PMID: 19801058 PMC3008558

[26] Stinear CM Barber PA Coxon JP Fleming MK Byblow WD. Priming the motor system enhances the effects of upper limb therapy in chronic stroke. Brain. (2008) 131:1381–90. doi: 10.1093/brain/awn051, PMID: 18356189

[27] Shiner CT Byblow WD McNulty PA. Bilateral priming before WII-based movement therapy enhances upper limb rehabilitation and its retention after stroke: a case-controlled study. Neurorehabil Neural Repair. (2014) 28:828–38. doi: 10.1177/1545968314523679, PMID: 24627333

[28] Stoykov ME Corcos DM Madhavan S. Movement-based priming: clinical applications and neural mechanisms. J Mot Behav. (2017) 49:88–97. doi: 10.1080/00222895.2016.1250716, PMID: 28277966 PMC6238643

[29] Wang RY Tseng HY Liao KK Wang CJ Lai KL Yang YR. rTMS combined with task-oriented training to improve symmetry of interhemispheric corticomotor excitability and gait performance after stroke: a randomized trial. Neurorehabil Neural Repair. (2012) 26:222–30. doi: 10.1177/1545968311423265, PMID: 21974983

[30] Grefkes C Fink GR. Connectivity-based approaches in stroke and recovery of function. Lancet Neurol. (2014) 13:206–16. doi: 10.1016/S1474-4422(13)70264-3, PMID: 24457190

[31] Liu Y Zhang L Zhang X Ma J Jia G. Effect of combined vagus nerve stimulation on recovery of upper extremity function in patients with stroke: a systematic review and meta-analysis. J Stroke Cerebrovasc Dis. (2022) 31:106390. doi: 10.1016/j.jstrokecerebrovasdis.2022.106390, PMID: 35334250

[32] Kimberley TJ Prudente CN Engineer ND Pierce D Tarver B Cramer SC. Study protocol for a pivotal randomised study assessing vagus nerve stimulation during rehabilitation for improved upper limb motor function after stroke. Eur Stroke J. (2019) 4:363–77. doi: 10.1177/2396987319855306, PMID: 31903435 PMC6921938

[33] Engineer ND Kimberley TJ Prudente CN Dawson J Tarver WB Hays SA. Targeted vagus nerve stimulation for rehabilitation after stroke. Front Neurosci. (2019) 13:280. doi: 10.3389/fnins.2019.00280, PMID: 30983963 PMC6449801

[34] Fischer HC Triandafilou KM Thielbar KO Ochoa JM Lazzaro EDC Pacholski KA. Use of a portable assistive glove to facilitate rehabilitation in stroke survivors with severe hand impairment. IEEE Trans Neural Syst Rehabil Eng. (2016) 24:344–51. doi: 10.1109/TNSRE.2015.2513675, PMID: 26731772

[35] Thielbar KO Triandafilou KM Fischer HC O’Toole JM Corrigan ML Ochoa JM. Benefits of using a voice and EMG-driven actuated glove to support occupational therapy for stroke survivors. IEEE Trans Neural Syst Rehabil Eng. (2017) 25:297–305. doi: 10.1109/TNSRE.2016.2569070, PMID: 27214905

[36] Byblow WD Stinear CM Smith MC Bjerre L Flaskager BK McCambridge AB. Mirror symmetric bimanual movement priming can increase corticomotor excitability and enhance motor learning. PLoS One. (2012) 7:e33882. doi: 10.1371/journal.pone.0033882, PMID: 22457799 PMC3310871

[37] Siebner HR. A primer on priming the human motor cortex. Clin Neurophysiol. (2010) 121:461–3. doi: 10.1016/j.clinph.2009.12.009, PMID: 20064742

[38] Kloek CJJ Bossen D Spreeuwenberg PM Dekker J de Bakker DH Veenhof C. Effectiveness of a blended physical therapist intervention in people with hip osteoarthritis, knee osteoarthritis, or both: a cluster-randomized controlled trial. Phys Ther. (2018) 98:560–70. doi: 10.1093/ptj/pzy045, PMID: 29788253 PMC6016690

[39] Koppenaal T Pisters MF Kloek CJ Arensman RM Ostelo RW Veenhof C. The 3-month effectiveness of a stratified blended physiotherapy intervention in patients with nonspecific low back pain: cluster randomized controlled trial. J Med Internet Res. (2022) 24:e31675. doi: 10.2196/31675, PMID: 35212635 PMC8917429

